# Anaphylaxis due to progesterone hypersensitivity: A rare cause of recurrent anaphylaxis

**DOI:** 10.5415/apallergy.0000000000000247

**Published:** 2025-11-10

**Authors:** Van Khanh Bui, Thi Trinh Cao, Hong Ngoc Nguyen

**Affiliations:** 1 Allergy and Clinical Immunology Department, Bach Mai Hospital, Hanoi, Vietnam; 2 Allergy and Clinical Immunology Department, Hanoi Medical University, Hanoi, Vietnam; 3 Allergy and Clinical Immunology Department, Quang Ninh General Hospital, Quang Ninh, Vietnam

**Keywords:** Anaphylaxis, case report, intradermal test, progesterone hypersensitivity, recurrent anaphylaxis

## Abstract

Progesterone hypersensitivity (PH) is a rare but underrecognized cause of recurrent anaphylaxis in women, particularly those of reproductive age. Both endogenous and exogenous progesterone can trigger hypersensitivity reactions, with clinical presentations ranging from urticaria to life-threatening anaphylaxis. We report a case of a 33-year-old Vietnamese woman who experienced six episodes of unexplained anaphylaxis over two years. A thorough clinical history revealed exposure to intramuscular progesterone during in vitro fertilization cycles and cyclical vulvar skin eruptions before menstruation. Skin prick testing with injectable progesterone was negative, while intradermal testing produced a positive reaction, confirming the diagnosis of PH. The patient was thoroughly counseled regarding her condition and equipped with an epinephrine auto-injector alongside a written anaphylaxis action plan. Daily antihistamine therapy with levocetirizine was initiated to mitigate the risk of endogenous progesterone-induced reactions. Given her stable condition and sterilization history, no further hormonal intervention was required. During two years of follow-up, she remained asymptomatic, with no recurrence of anaphylaxis. This case highlights the importance of considering PH in the differential diagnosis of recurrent anaphylaxis in women, especially those with cyclical allergic symptoms or a history of exogenous progesterone exposure. Early recognition and appropriate management are crucial to prevent potentially life-threatening events.

## 1. Introduction

Anaphylaxis is a severe, potentially life-threatening systemic hypersensitivity reaction with diverse triggers. In a significant proportion of cases, particularly among women, the cause of anaphylaxis remains unidentified and is classified as idiopathic. Progesterone hypersensitivity (PH), although rare, is an important but often overlooked cause of recurrent anaphylaxis in women of reproductive age [1]. PH can result from sensitization to endogenous or exogenous progesterone. It may present with a wide spectrum of allergic manifestations, ranging from cutaneous symptoms to severe systemic reactions, including anaphylaxis [2].

We present a rare case of recurrent idiopathic anaphylaxis later diagnosed as PH, emphasizing the importance of detailed history, appropriate diagnostic testing, and consideration of PH in the differential diagnosis of unexplained anaphylaxis.

## 2. Case presentation

### 2.1. Patient information

A 33-year-old Vietnamese woman was referred to the Allergy and Clinical Immunology Department of Bach Mai Hospital for outpatient evaluation following six episodes of unexplained anaphylaxis between 2022 and 2024. The first five episodes were classified as grade II reactions, presenting with generalized urticaria, dyspnea, and abdominal discomfort, without documented hypotension. The sixth and most recent episode, occurring four weeks before presentation, was more severe and involved urticaria, periorbital angioedema, abdominal pain, shortness of breath, dizziness, and hypotension. She was treated successfully with intramuscular epinephrine and supportive care at a local hospital.

She denied any known drug, food, insect sting, or Non-Steroidal Anti-Inflammatory Drug (NSAID) allergies. Her gynecologic history included three in vitro fertilization (IVF) cycles between 2015 and 2017, during which she received daily intramuscular progesterone (75 mg) for luteal phase support. Since her first pregnancy in 2015, the patient had experienced recurrent episodes of pruritic erythematous papules localized to the vulvar area, consistently appearing 2–3 days before menstruation and resolving spontaneously shortly after the onset of menses. These episodes were not associated with abnormal vaginal discharge or other gynecologic symptoms. Although no clear temporal relationship was established between the anaphylactic episodes and progesterone exposure, the recurrent cyclic skin symptoms and history of exogenous hormone use prompted further evaluation for PH. At the time of clinical assessment, no active skin lesions were observed. The patient had undergone sterilization following her last spontaneous pregnancy in 2021.

### 2.2. Clinical findings

During the sixth episode, the lowest recorded blood pressure was 80/50 mmHg, measured before epinephrine administration at a local facility. Upon evaluation at our center four weeks later, the patient was asymptomatic and hemodynamically stable. Vital signs were as follows: blood pressure 100/60 mmHg, heart rate 100 bpm, respiratory rate 20 breaths per minute, oxygen saturation 97% on room air, and temperature 37°C. Physical examination was unremarkable, with no rash, urticaria, or angioedema observed.

### 2.3. Diagnostic assessment

Initial laboratory investigations, including complete blood count, liver and renal function tests, serum electrolytes, and total IgE, were within normal limits. Chest radiography and abdominal ultrasound revealed no abnormalities.

**Figure 1.**
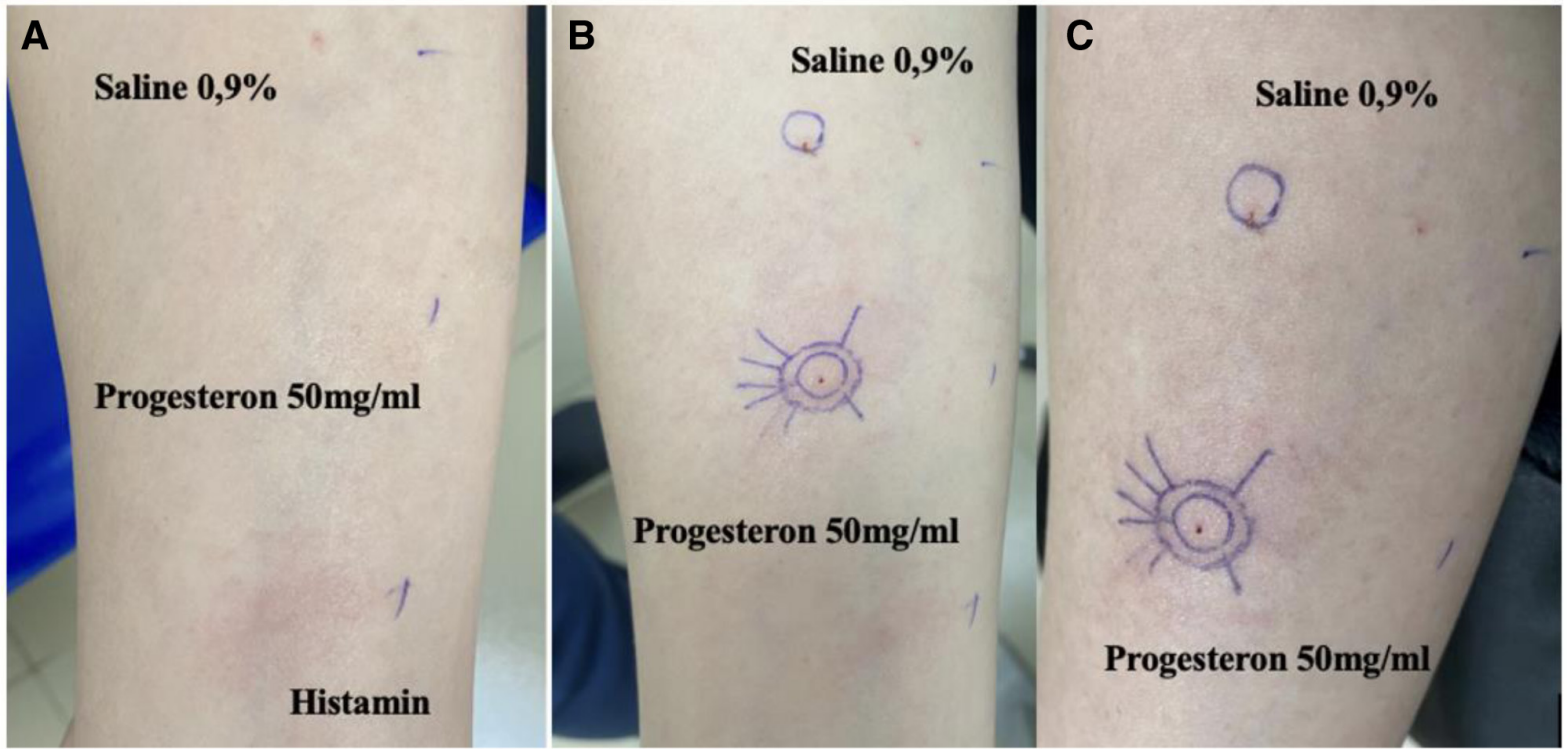
Skin test results with injectable progesterone, from left to right: (A), negative skin prick test; (B), positive intradermal test after 15 minutes, showing a 6 mm wheal with erythema; and (C) persistent positive intradermal test after 30 minutes, confirming progesterone hypersensitivity.

Skin prick testing was performed four weeks after the patient’s most recent anaphylactic episode. At the time of testing, she had discontinued corticosteroids and antihistamines for three weeks. Testing included common aeroallergens, food allergens, and injectable progesterone (50 mg/mL, standard protocol), all of which yielded negative results. The positive histamine control produced an appropriate wheal-and-flare response (Fig. 1A).

Subsequently, an intradermal test was performed using the same progesterone preparation diluted 1:100. A 6 mm wheal with surrounding erythema developed at 15 minutes (Fig. 1B) and persisted at 30 minutes (Fig. 1C), consistent with a positive reaction. No response was observed at the negative control site or in healthy female controls, supporting the specificity of the reaction.

Differential diagnoses including carcinoid syndrome, pheochromocytoma, and systemic mastocytosis were excluded through clinical and laboratory evaluation. Collectively, these findings supported the diagnosis of PH.

### 2.4. Therapeutic interventions

During the acute episode, the patient received intramuscular epinephrine, intravenous fluids, and antihistamines at a local hospital, resulting in symptom improvement. Upon transfer to our center, no additional pharmacologic treatment was required.

After confirming PH, the patient was counseled regarding her condition and educated about the risk of future anaphylaxis. As she no longer required exogenous progesterone, no immediate hormonal intervention was necessary.

To minimize the risk of anaphylaxis due to endogenous progesterone fluctuations, daily levocetirizine (5 mg) was prescribed. She was provided with self-injectable epinephrine and trained on its use. Hormonal suppression and omalizumab were discussed as options if symptoms recurred, but were deferred based on her preference and stable condition.

### 2.5. Follow-up and outcomes

The patient was followed with regular monthly evaluations. Over a two-year follow-up period, no recurrence of anaphylaxis or other allergic symptoms occurred. She adhered to daily antihistamine therapy, and notably, the premenstrual vulvar pruritic eruptions she had experienced since 2015 also resolved completely. She demonstrated appropriate preparedness for anaphylaxis management and reported significant improvement in quality of life.

## 3. Discussion

PH is an uncommon but clinically significant disorder characterized by hypersensitivity reactions to endogenous or exogenous progesterone. First described in 1921, PH has been increasingly recognized in recent decades, although fewer than 200 cases have been reported to date, suggesting significant underdiagnosis in clinical practice [3, 4]. The clinical manifestations of PH are heterogeneous, ranging from isolated cutaneous eruptions such as urticaria, vesicular rashes, or erythema multiforme-like lesions to severe systemic reactions, including asthma exacerbations and life-threatening anaphylaxis [5, 6]. In many cases, symptoms exhibit a cyclical pattern, correlating with the luteal phase of the menstrual cycle when endogenous progesterone levels peak. Exogenous progesterone exposure, such as during IVF, hormonal therapy, or contraceptive use, may also trigger sensitization and subsequent allergic reactions [6, 7]. The underlying immunopathogenesis of PH remains incompletely understood and appears to be multifactorial. Immediate-type (IgE-mediated) hypersensitivity mechanisms are supported by positive skin testing and mast cell-driven symptoms [3, 5, 8]. However, delayed-type (T-cell mediated) and immune complex-mediated pathways have also been proposed, accounting for the broad clinical spectrum observed in PH [5, 8]. Notably, structurally modified exogenous progestogens, such as 19-nortestosterone derivatives or 17α-hydroxyprogesterone analogs, may carry a higher risk of sensitization than natural progesterone [7]. Additionally, the immunomodulatory effects of sex hormones, including progesterone, further complicate disease pathogenesis, as they can influence both innate and adaptive immune responses [9]. Diagnosing PH remains challenging due to its rarity, diverse presentations, and overlap with other conditions such as NSAID hypersensitivity, chronic spontaneous urticaria, estrogen hypersensitivity, and catamenial anaphylaxis [6, 10]. Diagnosis is primarily based on clinical suspicion, with a compatible history of cyclical allergic symptoms, unexplained anaphylaxis, or known exogenous progesterone exposure serving as key clues. Skin testing with progesterone, including skin prick and intradermal testing, provides supportive evidence when interpreted in the appropriate clinical context [4, 8]. However, these tests lack standardization, and their sensitivity and specificity remain variable. Our case highlights several important aspects of PH diagnosis and management. First, the patient presented with recurrent unexplained anaphylaxis over two years, a common but often overlooked manifestation of PH [6, 11]. Her prior IVF cycles involving exogenous progesterone likely contributed to sensitization, a phenomenon also reported in the literature [6, 7]. The positive intradermal test with injectable progesterone was instrumental in confirming the diagnosis, underscoring the value of thorough history-taking and targeted diagnostic evaluation.

Management of PH is highly individualized, depending on symptom severity, reproductive status, patient preference, and treatment availability. Options range from antihistamines for mild cutaneous symptoms to more aggressive interventions such as hormonal suppression with gonadotropin-releasing hormone agonists, tamoxifen, or danazol for severe or refractory cases [6]. Desensitization protocols with gradually increasing progesterone doses have been explored, particularly for women desiring pregnancy [6, 11]. Biologic therapies, notably omalizumab, an anti-IgE monoclonal antibody, have shown promise in select patients with progesterone-induced anaphylaxis or refractory PH, as demonstrated by Heffler et al. [11].

In our case, given the patient’s sterilization status, stable clinical condition, and absence of ongoing exogenous progesterone exposure, a conservative approach consisting of daily antihistamines and an anaphylaxis action plan was deemed appropriate. During two years of follow-up, she remained symptom-free, demonstrating the potential effectiveness of noninvasive management in carefully selected patients.

Despite growing awareness, PH remains underrecognized and likely underdiagnosed, especially in resource-limited settings. Increased clinician awareness, particularly among allergists, gynecologists, and emergency physicians, is essential for timely diagnosis and prevention of potentially life-threatening complications. Moreover, further research is warranted to standardize diagnostic protocols and establish evidence-based management guidelines for this rare but impactful condition.

## 4. Conclusions

PH is a rare but potentially life-threatening condition that should be considered in women with recurrent, unexplained anaphylaxis or cyclical allergic symptoms. Early recognition relies on a high index of suspicion, thorough history-taking, and appropriate diagnostic evaluation, especially in those with exogenous progesterone exposure. Management should be individualized based on symptom severity and reproductive status, with options ranging from antihistamines to hormonal suppression, desensitization, or biologic therapies. This case emphasizes the need to include PH in the differential diagnosis of recurrent anaphylaxis and highlights the importance of a personalized, multidisciplinary approach to optimize patient outcomes.

## Conflicts of interest

The authors have no financial conflicts of interest.

This case report was conducted in accordance with institutional and international ethical standards. Institutional review board approval was not required for single case reports at our institution. Written informed consent for publication was obtained from the patient.

## Author contributions

We confirm that all authors contributed equally to the preparation of this manuscript, including the conception of the case, data collection, analysis, and manuscript drafting and revision. All authors have read and approved the final version of the manuscript.
